# Orofacial pain in juvenile idiopathic arthritis is associated with stress as well as psychosocial and functional limitations

**DOI:** 10.1186/s12969-019-0385-7

**Published:** 2019-12-19

**Authors:** Alexandra Dimitrijevic Carlsson, Kerstin Wahlund, Erik Kindgren, Thomas Skogh, Carin Starkhammar Johansson, Per Alstergren

**Affiliations:** 1 Orofacial Pain and Jaw Function, Malmö University, Malmö, Sweden; 2 Center for Oral Rehabilitation in Linköping, and Department of Biomedical and Clinical Sciences, Linköping University, Linköping, Sweden; 3 Scandinavian Center for Orofacial Neurosciences, Malmö, Sweden; 4 Department of Stomatognathic Physiology, Kalmar County Hospital, Kalmar, Sweden; 5 Department of Pediatrics, Västervik Hospital, Västervik, Sweden; 6 Division of Pediatrics, Department of Clinical and Experimental Medicine, Linköping University, Linköping, Sweden; 7 Department of Pediatrics, Skaraborg Hospital, Skövde, Sweden; 8 Division of Neuro and Inflammation Sciences, Department of Clinical and Experimental Medicine, Linköping University, Linköping, Sweden; 9 Skåne University Hospital, Specialized Pain Rehabilitation, Lund, Sweden; 10 Orofacial Pain Unit, Malmö University, Malmö, Sweden

**Keywords:** Adolescents, Children, Juvenile idiopathic arthritis, Orofacial pain, Psychosocial, Stress Temporomandibular joint disorders

## Abstract

**Background:**

The aim of this study was to investigate relations between psychosocial factors, signs and symptoms of orofacial pain and jaw dysfunction in patients with juvenile idiopathic arthritis (JIA).

**Methods:**

Forty-five patients with JIA (median age 12 years) and 16 healthy matched controls (median age 13 years) were examined according to the diagnostic criteria for temporomandibular disorders (DC/TMD). The subjects answered the DC/TMD questionnaires regarding psychosocial factors (pain intensity, pain–related disability, depression, stress, catastrophizing, pain locations and jaw function).

**Results:**

JIA patients with orofacial pain had higher degree of stress, depression, catastrophizing and jaw dysfunction compared to subjects without. In turn, these factors were associated with orofacial pain intensity. Also, patients with orofacial pain had higher systemic inflammatory activity.

**Conclusions:**

Orofacial pain in patients with JIA is associated with stress, psychological distress, jaw dysfunction and loss of daily living activities. Pain intensity seems to be the major pain aspect related to these factors. In addition, systemic inflammatory activity appears to be an important factor contributing to orofacial pain in JIA.

## Background

Juvenile idiopathic arthritis (JIA) is defined as arthritis of unknown origin with onset before the age of 16 and persisting for at least 6 weeks. JIA is categorized into seven subtypes: systemic arthritis, oligoarthritis, polyarthritis (rheumatoid-factor-negative, rheumatoid-factor-positive), psoriatic arthritis, enthesitis-related arthritis and undifferentiated arthritis [1]. JIA is a systemic inflammatory disease that primarily affects the musculoskeletal system but also other organs like the eyes may become affected with local inflammation. The disease is characterized by autoimmune reactions often targeting synovial tissues, resulting in chronic arthritis [2, 3].

JIA can involve any joint, including the temporomandibular joints (TMJ), but symptoms and signs may vary. The prevalence of JIA is estimated to be 56 individuals per 100,000 in the Swedish population. The estimated annual incidence is 12 cases per 100,000 [4]. These children and adolescents have chronic or recurrent pain and disability, which may severely limit their functional ability, impact growth and impair well-being [5, 6]. The children may refrain from school education and withdraw from social activities due to functional limitations and pain [5]. JIA pain may cause these children a sense of being misunderstood and stigmatized. Children with JIA are also at increased risk of developing depression [6]. Psychosocial stress and functional dependence in children with chronic illness are risk factors for poor coping [5]. Children with JIA, and other chronic diseases, are at increased risk of chronic musculoskeletal pain [7].

Forty to 96 % of children with JIA develop TMJ arthritis [8]. TMJ pain is, however, rare but stiffness, crepitus and restricted maximal mouth opening capacity are often reported [9, 10]. TMJ arthritis may result in mandibular micrognathia due to mandibular growth inhibition as well as articular bone tissue and cartilage destruction, causing posterior rotation of the mandible and resulting in frontal open bite [11, 12].TMJ arthritis may also result in persistent or recurrent pain.

The aim of this study was to investigate whether pain and function of the masticatory system, including the TMJ, are related to psychosocial factors and systemic inflammatory activity in patients with JIA.

## Methods

### Subjects

This case-control cross-sectional study was conducted at the Centre of Oral Rehabilitation in Linköping, Sweden between 2015 and 2018. Forty-five JIA patients aged between 6 and 16 years (33 girls and 12 boys) were included (Table 1). The JIA patients were referred consecutively from four pediatric departments in South-east Sweden (Linköping university hospital, Vrinnevi Hospital/Norrköping, Motala Hospital and Västervik Hospital). Inclusion criteria were JIA-diagnosis according to the criteria of the International League of Association for rheumatology (ILAR) [1]. Exclusion criteria were diabetes, inflammatory-bowel disease, other chronic pain condition than JIA and psychiatric disease (depression and anxiety were, however, allowed due to the frequent occurrence and important role in chronic pain).

**Table 1:**
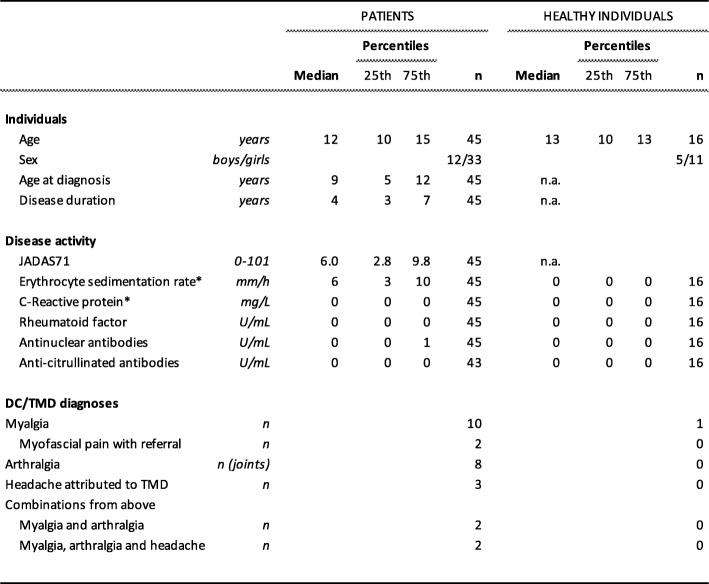
Demographic data, disease activity and temporomandibular disorder diagnoses for 45 patients with juvenile idiopathic arthritis and 16 age- and sex-matched healthy individuals

Nineteen (43%) of the patients had oligoarthritis, 15 (33%) had polyarthritis and 11 (24%) other subtypes of JIA (systemic arthritis, psoriatic arthritis, enthesitis-related arthritis and undifferentiated arthritis). The patient sample in the present study was representative for JIA patients in Scandinavia [13]. At inclusion, 34 patients had anti-rheumatic therapy; 28 with methotrexate, 12 with biologics (adalimumab, etanercept) and seven with prednisolone. The patients without antirheumatic therapy were on NSAIDs or in remission. In our study population, the median 71-joint Juvenile Arthritis Disease Activity Score (JADAS71) was found to be 6.0. JADAS71 over 4.0 is considered to show high disease activity [14] (Table 1).

Sixteen healthy age- and sex- matched individuals were recruited from the public dental health clinic in Linköping, Sweden. Inclusion criteria were healthy age- and sex- matched individuals with no subjective pain from the orofacial region. Exclusion criteria were rheumatic disease, diabetes, inflammatory-bowel disease, other chronic pain conditions and psychiatric disease (depression and anxiety were allowed).

### Clinical examination

The diagnostic criteria for temporomandibular disorders (DC/TMD) [15] was used to diagnose the patients. The DC/TMD comprises two domains, a physical Axis I (clinical condition) and a psychosocial Axis II (psychosocial distress). The applied clinical examination corresponds very well to the consensus-based recommendations for clinical orofacial examination in JIA, except for examination of craniofacial deformations [16].

The clinical examination for Axis I diagnostics requires a pain history, assessed by a questionnaire, and a well-defined and structured clinical examination. Clinical assessments evaluate familiar pain localizations, jaw movement capacity (lateral, protruding, and mouth opening), familiar jaw movement pain, TMJ noises and familiar pain upon palpation of the masticatory muscles and TMJ. The criteria for DC/TMD Axis I diagnoses are validated from 18 years of age [15] and comprise TMJ arthralgia, masticatory muscle myalgia, headache attributed to TMD, degenerative joint disease and TMJ disk displacements. Multiple diagnoses are allowed in DC/TMD.

The DC/TMD Axis II assesses the patient’s psychosocial function and distress as well as pain-related disability. Axis II is based on validated instruments (questionnaires) and interpretation guidelines. It includes instruments for assessing pain behavior, jaw function, and psychosocial functioning and distress [15].

All patients and healthy individuals were examined by one dentist (ADC) calibrated in the clinical and research use of DC/TMD by the DC/TMD Training and Calibration Center at the Department of Orofacial Pain and Jaw Function, Faculty of Odontology, Malmö University, Sweden.

All the participants completed the questionnaires before clinical examination. The participants 12–16 years of age answered all questions.

### Psychosocial status

Pain intensity and pain-related disability were assessed with the Graded Chronic Pain Scale (GCPS). GCPS is a reliable seven-item questionnaire that assesses pain intensity and pain-related disability [17]. The two GCPS subscales used in the present study were: characteristic pain intensity (mean of pain intensity of current pain, average pain during the last week and the worst pain during the last week scored on separate 0–10 scales with end-points “no pain” and “worst pain imaginable”) and pain-related disability (mean of how much the pain has changed the patient’s ability to take part in daily activities, in recreational, family and social activities and school on separate 0–10 scales with end-points “No change” and “Extreme change”). The questions in the GCPS have been used in a previous study in adolescents aged 10–19 years [18].

Jaw function limitation was assessed using the Jaw Functional Limitation Scale (JFLS-8). JFLS-8 contains eight items regarding jaw function (jaw mobility, mastication, and verbal and emotional expression) that the patient grades separately on a 11- point scale (from no limitation to severe limitation) [19]. JFLS-14 has been used in previous studies in adolescents aged 12–19 years [18].

The four-item Patient Health Questionnaire (PHQ–4) was used to assess the degree of symptoms from depression and anxiety. There are established cut-off limits for “normal”, “mild”, “moderate” and “severe” degree of symptoms from depression and anxiety [20, 21]. The PHQ-4 questionnaire has been validated in the general population, starting from the age of 14 [21]. In another study the PHQ-4 questionnaire was used to detect psychosocial impact (depression and anxiety) after otoplasty (ear malformations) surgery in children age 8–17 [22].

The Pain Catastrophizing Scale (PCS) was used to assess degree of catastrophizing [23]. PCS includes 13 items rated on a five-point scale ranging from 0 = “not at all true” to 4 = “very true.” The items are divided across three subscales: rumination, magnification and helplessness. The PCS has been validated in children aged 8–16 years in community samples as well as in children with chronic pain [24–26].

The 10-item Perceived Stress Scale (PSS-10) [27, 28] designed by Cohen et al. was used to assess the degree of stress. Higher scores reflect greater levels of perceived stress. The Swedish version of PSS-10 has been shown to have good reliability and construct validity [29]. The PSS has been used for adolescents (15–19 years) in a previous study [30].

The disease-specific Childhood Health Assessment Questionnaire (CHAQ) was used to assess functional ability in daily life activities among children with JIA. CHAQ is designed to capture the physical and psychosocial well-being of children with JIA. CHAQ is a reliable and valid tool and it comprises 30 questions [31, 32].

The body drawing in the McGill Pain Questionnaire was used to record pain locations. In this study, we distinguished between local (orofacial area), regional (orofacial area and neck/shoulder areas) and widespread pain (orofacial area, neck/shoulder and the rest of the body) [33, 34].

The Juvenile Arthritis Disease Activity Score (JADAS) is a composite disease activity score specific to JIA [35]. It is simple to calculate using four variables measured in the clinical setting: active joint count, physician global assessment, parent global evaluation and erythrocyte sedimentation rate. JADAS has been shown to be feasible, with good construct validity, discriminant validity and responsiveness to clinically important change [35].

### Statistical analyses

Non-parametric statistics were used. For descriptive statistics, median and 25th/75th percentiles were reported. For analytical statistics, the Mann-Whitney U-test was used to calculate the significance of differences between groups and the Spearman ranked correlation coefficient was used to calculate the significance of correlations between variables. A probability level of *P* < 0.05 was considered as significant.

## Results

### Patients and healthy individuals

Table 2 shows the clinical and psychosocial variables in the patients as well as the healthy individuals. There was no significant difference between the patients and healthy individuals regarding any of the clinical and psychosocial variables assessed in both groups.

**Table 2:**
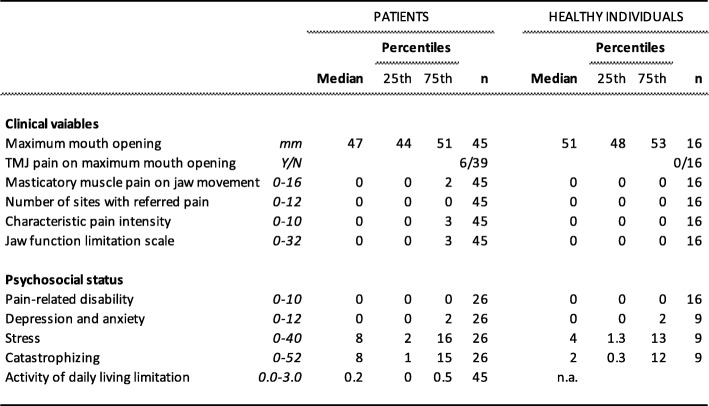
Clinical and psychosocial data from 45 patients with juvenile idiopathic arthritis and 16 age- and sex-matched healthy individuals

Regarding pain locations and distribution, two (4%) of the patients had local pain distribution, 35 patients (78%) had generalized pain distribution and 8 (18%) patients did not report any pain location. Of the patients with generalized pain distribution, 46% had pain in the masticatory system as well. None of the healthy individuals indicated any self-reported pain.

Five (26%) of the patients with oligoarthritis and ten (71%) of the patients with polyarthritis had orofacial pain.

### Relation between clinical and psychosocial variables in the patients

**Fig. 1:**
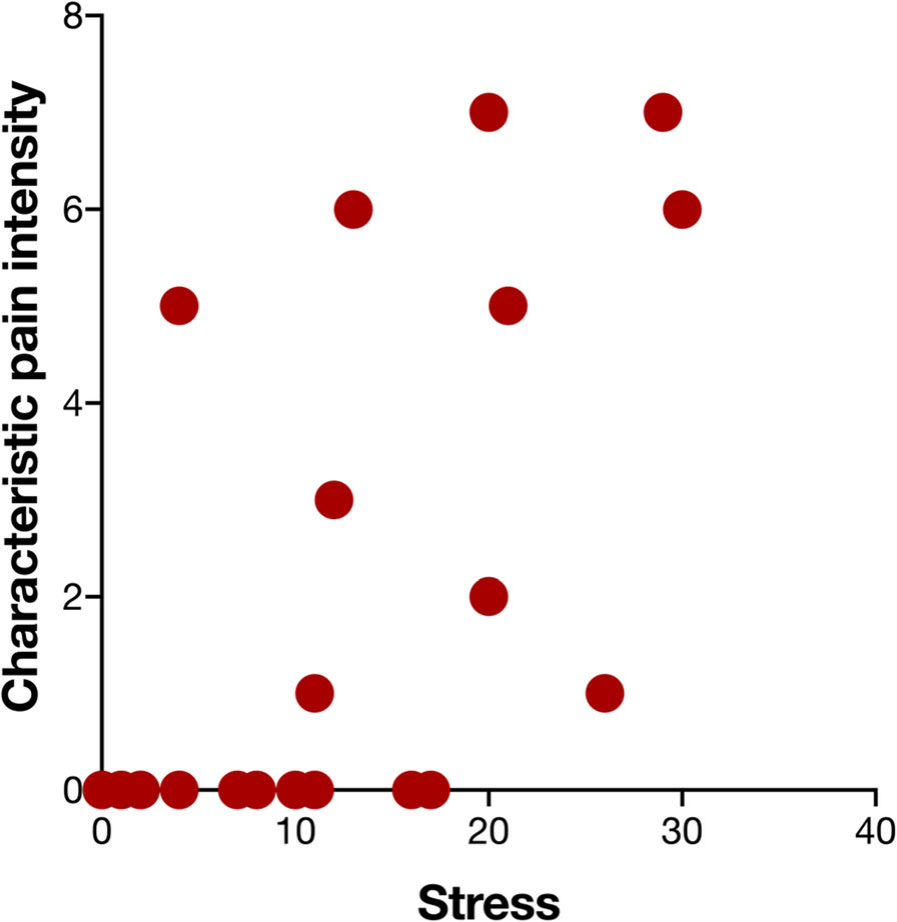
Scatter-plot showing the relation between degree of stress, as assessed by the Perceived Stress Scale-10, and characteristic orofacial pain intensity in 45 patients with juvenile idiopathic arthritis (Spearman’s ranked correlation test: r_s_ = 0.70, *n* = 45, *p* < 0.001)

Table 3 shows the significant correlations between the clinical and psychosocial variables in the JIA patient group. Figure 1 shows the relation between perceived stress and characteristic pain intensity. Among patients scoring perceived stress higher than “normal” (PSS-10 score 0–12), eight out of ten had self-reported orofacial pain (characteristic pain intensity; Fig. 1). Of these, five had a characteristic pain intensity > 5.

**Table 3:**
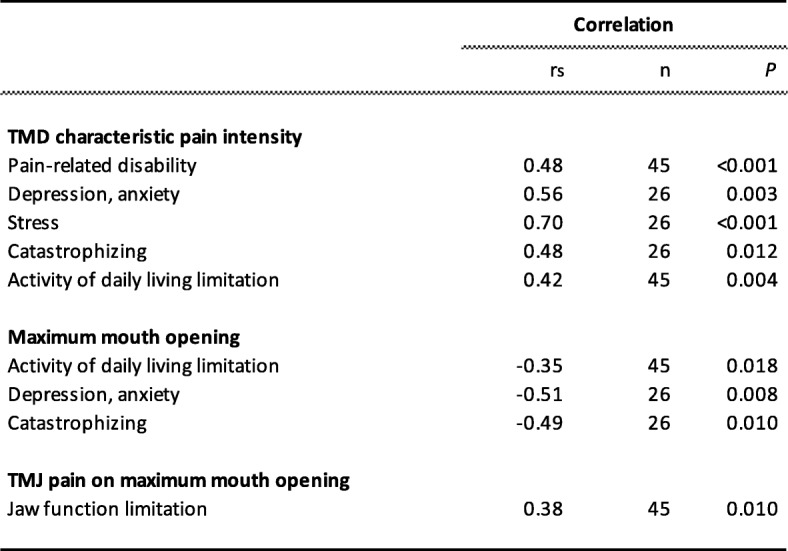
Significant correlations between clinical and psychosocial variables in 45 patients with juvenile idiopathc arthritis

Table 4 shows the clinical and psychosocial variables in the 45 JIA patients with or without orofacial pain. Neither depression/anxiety nor catastrophizing reached a significant difference between patients with or without orofacial pain.

**Table 4:**
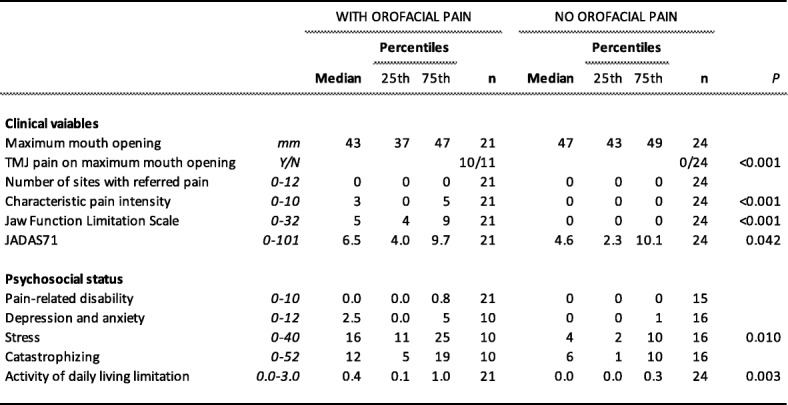
Clinical and psychosocial variables in 45 patients with JIA with and without orofacial pain (masticatory muscle myalgia, temporomandibular joint (TMJ) arthralgia, TMJ pain on jaw movement and function)

## Discussion

This study indicates that orofacial pain in patients with JIA is associated with stress, psychological distress, jaw dysfunction and loss of daily activities. Pain intensity seems to be the major pain aspect related to these factors. In addition, systemic inflammatory activity seems to be an important factor contributing to orofacial pain in JIA. Myalgia is probably a major reason for orofacial pain in these patients. When assessing the masticatory system in JIA and planning treatment, it seems to be of particular importance to also consider stress and systemic inflammatory activity.

### Orofacial pain and psychosocial factors

In the JIA patients, orofacial pain intensity and perceived stress were related in our patient sample, i.e. stress scores were higher in patients with orofacial pain compared to those without and there was a strong relation between degree of stress and orofacial pain intensity in these patients. Also pain-related disability, distress and catastrophizing were associated with orofacial pain intensity even though there was no difference between JIA patients with or without orofacial pain.. TMJ pain among JIA patients is neither common in general [9] nor in our patients. Instead, myalgia of the masticatory muscle system was found to be common in these patients. Abramowicz et al. 2013 found a substantial overlap between the diagnoses TMJ arthritis and masticatory muscle myofascial pain in JIA [36]. It therefore seems important to consider that masticatory muscle pain in JIA patients is to some extent related to the disease and therefore warrants to be included in the overall assessment of JIA patients.

On the other hand, adolescent JIA patients have been found to experience equivalent levels of anxiety and depressive symptoms as healthy adolescents, as also found in our study. For adolescent JIA patients, anxiety and depressive symptoms have been associated with pain and disability but not with inflammation [37].

Stress and chronic pain in the general adolescent population have been found to be related. For example, both musculoskeletal pain and perceived stress appears prevalent among 15- and 16-year adolescents [38]. Pain intensity seems to be equally related to stress in adolescent girls and boys, whereas girls reports higher levels of stress, higher pain intensity and more headache compared to the boys [38]. Stress seems to be related to orofacial pain in this study even though stress is common among adolescents. Although stress among adolescents in general is common, it is not always related to pain. This is exemplified by the JIA patients that showed stress but did not experience pain in our study. However, elevated stress seems to be strongly associated with higher degrees of orofacial pain intensity.

The Perceived Stress Scale used in this study assesses how often subjects have found their lives unpredictable, uncontrollable and overloaded in the last month [29]. Elevated stress seems to be associated with a major impact on daily life, well-being and chronic pain [39]. Our results support this since a significant proportion of the patients seem to experience a severe impact on daily life and well-being from stress and pain. In addition, psychological stress may contribute to an imbalance in the immune response and thereby worsen the systemic inflammatory activity in JIA patients [40, 41].

The strong relation we found between stress and pain intensity support the findings by Österås et al. (2016) [38]. In that study, the pain entities with the strongest relations to stress were pain intensity and number of painful sites, although several other pain aspects (frequency, duration etc.) were investigated. The association between stress and pain most likely goes both ways, with chronic pain being one of the strongest stress factors with social, psychological and physical consequences [39, 42]. This further indicates that stress management and pain-reducing treatments should be combined in these patients.

### Orofacial pain versus activities in daily life and jaw function

There was an interesting relation between orofacial pain intensity and limitations in general functional ability during daily life activities like arising, dressing, walking, hygiene, use of aids/devices, reach and grip, as assessed by the CHAQ. CHAQ is certainly not specific for orofacial pain and our findings may therefore likely be due to the systemic inflammatory activity found in the JIA patients with orofacial pain. Our findings are supported by a study of RA patients, where there was a significant impact of TMJ pain and discomfort regarding activities of daily living, including physical exercise and jaw movements [43]. In that study, the impact of TMJ pain on daily activities was also related to inflammatory activity. In our study, CHAQ was used to assess functional ability in daily activities and it is commonly used for that purpose by Swedish pediatric rheumatologists [44]. Adequate treatment of orofacial pain in JIA therefore seems to be of great importance also for overall well-being. Future research and development may therefore warrant explorations on how to better incorporate orofacial pain assessment and therapy in pediatric rheumatology.

In the present study, consequences of TMJ pain on maximum mouth opening was related to limitation of jaw function, which is reasonable. Our results thus suggest that the limited jaw function is due to joint pain rather than internal derangements like disc displacement or fibrous adhesions. The maximum mouth opening capacity was not abnormally low at a group level in the patients, where 75% had a mouth opening capacity of 44 mm or more. This suggests that reduced jaw function, in some patients is not due to a limited mouth opening capacity but rather to chewing or communicative (for example smile, laugh and facial expressions) difficulties.

### Why orofacial pain in the JIA patients?

Less than half of the JIA patients, reported orofacial pain. Among these, pain in the masticatory muscles, including headache attributed to TMD, was more common than TMJ arthralgia. Only two of the healthy individuals reported orofacial pain.

A difficult question to answer is to what extent the orofacial pain is related to the JIA disease, or due to other factors, like masticatory muscle hypertension. JIA is a systemic inflammatory condition that not always will result in TMJ inflammatory activity, causing signs and symptoms from the jaw system. A common feature of JIA, and most rheumatologically disorders, is that pain, when present, is rarely limited to the joint(s) but is also located in the surrounding musculoskeletal system. This is most likely due to sensitization, peripheral and central, of the joints and adjacent areas [45] and contributes to pain, including orofacial pain.

The incidence of TMD pain in the general population has been found to rapidly increase from the age of 13–14 years [46, 47] in girls. TMD pain is uncommon before the age of 13. Most of the patients included in our study population were younger than 13–14 years, why the likely cause of the orofacial pain may be JIA. However, many children, particularly girls, develop other chronic pains that orofacial pains before the age of 13 [48].

### TMJ arthritis

JIA can cause TMJ arthritis and arthritis may cause pain, cartilage and bone tissue destruction and growth inhibition. There are no established diagnostic criteria for TMJ arthritis in JIA and contrast-enhanced magnetic resonance imaging is usually considered as the gold standard for assessing TMJ arthritis. The classical criteria for inflammation (swelling, redness, pain, warmth, loss of function) are not applicable regarding the TMJ since these are rare events in TMJ arthritis compared to other peripheral joints. Of these signs, TMJ pain is most common but still not common in JIA. The aim of this study did not include assessment of TMJ arthritis per se, but rather description and analysis of clinical and psychosocial factors related to JIA and orofacial pain. There is, however, an urgent need to develop clinical criteria to enable early identification of JIA patients with established or a risk for TMJ arthritis.

### Methodological considerations

The study sample of patients had a median systemic disease activity of 6.0 according to JADAS71. This was somewhat higher than the median national systemic disease activity in JIA, approximately 4.0, as reported to the Swedish pediatric JIA-register 2016 (http://barnreumaregistret.se) [49]. JADAS71 values over 4.0 corresponds to “substantial disease activity”. We still consider our sample to be representative both for the area, since we included most of the available JIA patients in the pediatric rheumatology clinics in Linköping, Norrköping, Motala and Västervik, Sweden, and regarding orofacial signs and symptoms since the pain (TMJ, muscle and movement) was similar in our sample compared to a recent JIA sample in Italy [50].

## Conclusions

This study indicates that orofacial pain in patients with JIA is associated with stress, psychological distress, jaw dysfunction and loss of daily living activities. Pain intensity seems to be the major aspect related to these factors. In addition, increased disease activity with more joint involvement seems to be an important factor contributing to orofacial pain in JIA. Myalgia, in addition to arthritis, seems to be an important source of orofacial pain in these patients.

## Data Availability

Data can be obtained from the corresponding author, Alexandra Carlsson: alexandra.carlsson@regionostergotland.se.

## Abbreviations

CHAQ: Childhood Health Assessment Questionnaire
DC/TMD: Diagnostic Criteria for Temporomandibular Disorders
GCPS: Graded Chronic Pain Scale
ILAR: International League of Association for rheumatology
JADAS 71: 71-joint Juvenile Arthritis Disease Activity Score
JFLS: Jaw Functional Limitation Scale
JIA: Juvenile Idiopathic Arthritis
PCS: Pain Catastrophizing Scale
PHQ–4: Patient Health Questionnaire
PSS-10: Perceived Stress Scale
TMJ: Temporomandibular Joint
